# Rootstock-specific bacterial microbiome and metabolome remodeling enhances glycine substitution efficacy for ammonium nitrate in watermelon

**DOI:** 10.3389/fpls.2025.1640174

**Published:** 2025-09-05

**Authors:** Zehao Chen, Tian Yao, Xingxing Bao, Yifei Wang, Shaowei Qiao, Luxue Tan, Hanbing Shi, Xinyi Chen, Ming Ding

**Affiliations:** College of Horticulture, Northwest A&F University, Xianyang, China

**Keywords:** glycine, ammonium nitrate, grafted watermelon, microbiome, bacterial metabolomics

## Abstract

**Introduction:**

Optimizing nitrogen sources and rootstock selection is crucial for sustainable watermelon production. However, the synergistic mechanisms between organic nitrogen forms and rootstocks remain poorly understood. This study investigates whether glycine, as an organic nitrogen source, modulates root-associated bacterial communities through rootstock-mediated effects to enhance watermelon growth.

**Methods:**

Grafted watermelon plants (scion: watermelon; rootstocks: self-grafted watermelon (CK), wild watermelon (T1), bottle gourd (T2), pumpkin (T3) were cultivated under glycine (G) or ammonium nitrate (A) treatments for 25 days. Plant growth, soil enzyme activity, rhizosphere bacterial communities (16S rRNA sequencing), and root metabolomes (UPLC–MS/MS) were analyzed.

**Results:**

Relative to ammonium nitrate, glycine to some extent increased bacterial α-diversity but there was no significant difference and altered β-diversity, whereas enhancing microbial network complexity. Rootstock genotype is the main driver of bacterial α diversity and shaped the bacterial network architecture: T1-supported networks exhibited strong associations enriched in two-component systems, whereas T3 networks reflected intensified resource competition. Rootstock identity also influenced root exudate profiles. T3 secreted high levels of amino acids and nucleotides with metabolic and defensive roles, correlating with the abundance of *Edaphobacter* and *Actinomadura*. In contrast, T1 increased *Acidibacter* abundance via lipid secretion. The rootstock–bacteria–metabolite interplay modulated soil enzyme activities, supported photochemical efficiency, and promoted biomass accumulation.

**Discussion:**

These findings demonstrate the potential of glycine as a sustainable nitrogen source and identify compatible scion–rootstock combinations that enhance rhizosphere microbial dynamics and plant performance. The study provides mechanistic insights into how root exudates shape bacterial community assembly, although further work is needed to elucidate the complexity of microbe–microbe interactions.

## 1 Introduction

The growth of terrestrial plants is regulated by soil microbial communities, which induce roots to secrete specific compounds (Anderson et al., 2024) Rhizosphere microorganisms and root exudates serve as sensitive indicators of plant growth status. Both the aboveground and belowground compartments of terrestrial plants harbor extensive microbial populations, with the gene abundance of rhizosphere microbial communities frequently exceeding that of the host plant. Accordingly, the microbiome is described as the “second plant genome” (Mendes et al., 2013). Substantial evidence demonstrates that the rhizosphere microbiome plays a critical role in plant health (Berendsen et al., 2012).

Watermelon (*Citrullus lanatus (Thumb.))*, a major cucurbit horticultural crop, is valued globally for its distinctive flavor and rich nutritional content (Guo et al., 2013) and is cultivated extensively. The industrialization and specialization of modern watermelon production, combined with the challenge of excessive fertilizer application worldwide, have led to adverse effects on plant growth and yield due to persistent continuous cropping and inappropriate nitrogen management (Hakeem et al., 2011). As a nitrogen-demanding crop, watermelon is particularly susceptible to nitrogen over-application (Nawaz et al., 2018). Excessive chemical fertilizer use in protected cultivation systems frequently leads to inefficient productivity, increased production costs, and aggravated soil pollution (Pang et al., 2017). Global efforts to reduce fertilizer usage have accelerated the transition from chemical to organic fertilizers in agriculture. The application of organic fertilizers is essential for improving soil organic nitrogen content; relative to chemical fertilizers, organic fertilizers release nutrients more gradually, provide greater persistence, and are less susceptible to leaching, thereby avoiding the need for repeated excessive application. Plants can directly acquire and utilize both inorganic and organic nitrogen forms (Murphy et al., 2000). Glycine, among the most abundant free amino acids in crop soils (Wang et al., 2014), is distinguished by its minimal molecular weight and simple structure, rendering it a promising substitute for inorganic nitrogen.

Grafting is a widely used agricultural practice, particularly in watermelon production, to mitigate the adverse effects of continuous cropping, enhance disease resistance, and improve mineral uptake, thereby supporting stable yields (Edelstein et al., 2011; Rivero et al., 2003). During cultivation, auto-toxic substances may accumulate in the soil; however, root replacement via grafting can alter the rhizosphere microbiota, promoting the degradation of such substances (Wang et al., 2023). The root systems of grafted plants have the capacity to recruit and reshape functional microbial communities, thereby maintaining plant health (Poudel et al., 2019), and enhancing the stability of microbial networks, with potential reductions in pathogen populations (Ruan et al., 2020).

Nevertheless, research on organic nitrogen fertilizers has predominantly focused on non-grafted crops (Zhang et al., 2022), and limited knowledge exists regarding the effect of fully substituting inorganic nitrogen with organic nitrogen on the rhizosphere microenvironment of grafted watermelon. In particular, the variations in root exudate composition and rhizosphere microbial community diversity across different rootstocks remain poorly characterized (Hassnin et al., 2023; Ling et al., 2013). Therefore, given the potential of glycine as an organic nitrogen source and the central role of rootstock–scion interactions, we hypothesized the following:

1. Glycine application would improve soil health, plant growth, and bacterial diversity in grafted watermelon by serving as a preferential carbon and nitrogen source for beneficial rhizobacteria, thereby enhancing nutrient use efficiency and reducing environmental effect compared to inorganic fertilizers.
2. The selection of rootstock genotype would significantly modulate the root exudate profile, facilitating the recruitment of a functionally beneficial rhizosphere bacterial microbiome that enhances plant resilience and productivity under glycine nutrition.
3. The synergistic interaction between root exudates (as shaped by rootstock) and glycine-enriched rhizosphere bacterial microbiomes would be a principal driver sustaining improvements in soil health and plant performance.

This study aims to evaluate the rhizosphere microecological environment and nutrient uptake characteristics, thereby providing theoretical and technical support for practical watermelon production.

## 2 Materials and methods

The experiment was conducted in a sunlight greenhouse at the Horticultural Field of Northwest A&F University’s North Campus, Yangling Demonstration Zone (34°17′31.297′′ N, 108°4′26.310′′ E).

### 2.1 Preparation of materials and nutrient solution

The watermelon scion variety ‘8424’ was procured from Xinjiang Mingxin Kehong Co., Ltd. Rootstock varieties included ‘Warrior’ wild watermelon (Citrullus colocynthis), ‘Qinkang Shui Gua’ bottle gourd (Lagenaria siceraria), and ‘Jingxin No.2’ white-seed pumpkin (Cucurbita moschata Duchesne), sourced from Hangzhou Yihe Seedling Co., Ltd., Hunan Xuefeng Seed Industry Co., Ltd., and the Beijing Academy of Agricultural Sciences, respectively.

Seeds were sterilized and pre-germinated prior to planting (Wen et al., 2020). After germination, uniform seedlings were randomly sown into 50-cell trays. Grafting was performed 15 days post-sowing for scions and 20 days post-sowing for rootstocks using the splice-grafting method described by (Oda, 1995). Following grafting, as outlined by (Kacjan-Maršić and Osvald, 2004), grafted plants were maintained at 28°C with relative humidity above 95% for 3 days, followed by gradual ventilation and light exposure. After a 10-day graft union healing period, plants were transplanted into pots containing a substrate mixture of peat, vermiculite, and perlite at a 3:1:1 volume ratio (10 cm × 10 cm × 5 cm). Experimental treatments were carried out in a controlled climate chamber with a photosynthetic photon flux density (PPFD) of 200 μmol m⁻² s⁻¹, a 16 h light/8 h dark photoperiod, culture temperatures of 28°C (day)/18°C (night), and 60% relative humidity.

The nutrient solution was prepared based on a modified half-strength Japanese Enshi formula for horticultural crops, with all nitrogen sources omitted to create a nitrogen-deficient baseline. Organic and inorganic nitrogen sources were then supplemented, with ammonium nitrate serving as the inorganic source and glycine as the organic source. Details of the nutrient solution composition are shown in **Table 1**.

**Table 1:** Nutrient solution formulation.

1/2 Nitrogen deficiency Japanese Enshi formula			Nitrogen forms	
Class	Fertilizer	Concentration (mg/L)	Ammonium nitrate	Glycine
			8mmol/L	
A	CaCl_2_	222.10	320.00 mg/L	600.20 mg/L
B	KH_2_PO_4_ MgSO_4_·7H_2_O	554.51 246.50		
C	MnSO_4_·H_2_O H_3_BO_3_ CuSO_4_·5H_2_O ZnSO_4_·7H_2_O NaMoO_4_·2H_2_O EDTA-FeNa	0.807 1.43 0.04 0.11 0.0137 10.00		

### 2.2 Experimental design and determination of physio-biochemical parameters

A randomized block design was employed, comprising four grafted rootstock treatments (watermelon, wild watermelon, bottle gourd, and pumpkin) and 2 nitrogen treatments (8 mmol/L ammonium nitrate and glycine), yielding 8 treatment combinations with nine plants each. Treatment details are presented in **Table 2**. The experiment spanned 25 days; each plant received 30 mL of nutrient solution daily and 100 mL of water every five days.

**Table 2:** Test treatment and numbering.

Test treatments	Nitrogen	Grafting combinations(S/R)
ACK AT1 AT2 AT3	Ammonium nitrate	Watermelon/Watermelon Watermelon/Wild watermelon Watermelon/Bottle gourd Watermelon/Pumpkin
GCK GT1 GT2 GT3	Glycine	Watermelon/Watermelon Watermelon/Wild watermelon Watermelon/Bottle gourd Watermelon/Pumpkin

Upon completion of the cultivation period, plants and substrates were separated. Rhizosphere soil was collected by gently shaking substrate from the roots using the method described by (Wollum, 1994), immediately stored on ice, and sieved through a 2 mm mesh. Portions of the sample were air-dried for soil enzyme activity analysis.

The aboveground and belowground plant parts were separated, weighed, dried at 65°C for 48 hours, and then reweighed. All samples were collected in three replicates, each consisting of three plants (3 × 3 = 9).

A portable modulation chlorophyll fluorometer (Model PAM2500, USA) was employed to determine the fluorescence yield parameters of the leaves. For chlorophyll content determination, 0.2 g of fresh leaves (excluding main veins) was extracted with 5 mL of acetone/ethanol (2:1, v/v). Absorbance was measured at 663 nm and 645 nm (Pérez-Patricio et al., 2018), and chlorophyll content was calculated according to (Gu et al., 2016):


Chlorophyll total (mg/g)=(8.2 * A663)+(20.2 * A645)

n the formula, A663 and A645 denote absorbance at 663 nm and 645 nm, respectively, with acetone/ethanol used as the calibration blank.

Air-dried substrate samples were used for soil enzyme activity measurement. Soil sucrase (S-SC) activity was measured using the sodium thiosulfate titration method (Gao et al., 2013); Urease activity was evaluated based on the concentration of the blue complex formed after treatment (Guo et al., 2012); Soil acid phosphatase (S-AP) activity was determined following the method of (Guan et al., 1986), utilizing toluene and disodium phenyl phosphate as assay reagents.

### 2.3 16S rRNA gene amplicon sequence processing

Upon completion of cultivation, plants and substrates were separated. Rhizosphere soil was collected by gently shaking the substrate from the roots using the method described by (Wollum, 1994), temporarily stored on ice, and immediately passed through a 2 mm mesh sieve. A portion of the sample was air-dried for soil enzyme activity assays, whereas another portion was stored at −80°C for microbial community analysis via 16S rRNA gene amplicon sequencing.

PCR amplification targeting the V4 region of the 16S rRNA gene was performed on extracted DNA samples using the 515F/806R primers (F: GTGCCAGCMGCCGCGGTAA; R: GGACTACHVGGGTWTCTAAT) (Walters et al., 2016). The reaction was conducted on a Bio-Rad T100 thermal cycler (Bio-Rad, USA) using the following protocol: each 30 μL reaction contained 15 μL Phusion Master Mix (2X; New England Biolabs), 0.2 μL each of forward and reverse primer (1 μM), approximately 10 ng template DNA, and ddH2O to final volume. Cycling conditions were 98°C for 10 min; 30 cycles of 98°C for 10 s, 50°C for 30 s, and 72°C for 30 s; followed by a final extension at 72°C for 5 min. PCR products were pooled in equimolar ratios according to concentration, mixed, and purified using 2% agarose gel electrophoresis. Target bands were recovered and further purified with a Qiagen Gel Extraction Kit (Qiagen, Germany) (Ahmed et al., 2021). Sequencing libraries were prepared using the TruSeq^®^ DNA PCR-Free Sample Preparation Kit (Illumina, San Diego, CA, USA). Library quality was assessed with a Qubit^®^ 2.0 Fluorometer (ThermoFisher Scientific, CA, USA) and Agilent Bioanalyzer 2100 (Agilent Technologies Inc., USA). Sequencing was performed on the Illumina NovaSeq 6000 platform.

For bacterial community analysis, raw 16S rRNA sequence data were quality-filtered using fastp (v0.19.6) (Chen et al., 2018), Paired-end reads were merged with FLASH v1.2.7, and clean data were further denoised in QIIME 2 using the Deblur algorithm (Knight et al., 2018) to generate high-quality amplicon sequence variants (ASVs). Taxonomic classification of ASVs was based on comparison to the SILVA reference database, with chimeric sequences removed (Haas et al., 2011). Microbial community α-diversity under different treatments was assessed by the Kruskal–Wallis test, whereas β-diversity differences between groups were evaluated using permutational multivariate analysis of variance (PERMANOVA). The Wilcoxon rank-sum test was applied to determine statistical significance of relative abundance changes at the ASV level between plant groups.

### 2.4 Metabolomic analysis

Separated plant root systems were thoroughly rinsed with deionized water and placed into shaded conical flasks containing 100 mL pure water for exudate collection. Following (Li et al., 2019), the collected root exudate solutions were incubated under light for 12 hours, filtered through a 0.22 μm membrane, freeze-dried (EPSILON 2–4 LSC, CHRIST, Germany), and stored at −80°C. Fresh leaves were collected at the same time.

Freeze-dried samples were reconstituted in 70% methanol containing an internal standard extraction solution at a 20× concentration ratio. The internal standard was prepared by dissolving 1 mg of standard in 1 mL of 70% methanol to yield a 1000 μg/mL stock solution, which was then diluted with 70% methanol to 250 μg/mL. After vortexing for 15 minutes and ice-water ultrasonication (KQ5200E) for 10 minutes, samples were centrifuged at 12,000 rpm and 4°C (5424R, Eppendorf) for 3 minutes. Supernatants were filtered through a 0.22 μm microporous membrane and stored in autosampler vials for subsequent UPLC–MS/MS analysis.

Sample preparation and metabolomics data processing were performed by Metware Biotechnology Co., Ltd. (Wuhan, China; https://www.metware.cn/). Instrumentation included an ultra-performance liquid chromatography (UPLC) system (ExionLCTM AD, http://sciex.com.cn/) and tandem mass spectrometry (MS/MS). Compound identification utilized the self-constructed Metware Database (MWDB) with secondary spectral information, removing isotope signals and redundant fragment ion signals from higher molecular weight compounds. Metabolite quantification was conducted using the multiple reaction monitoring (MRM) mode of triple-quadrupole mass spectrometry (Fraga et al., 2010).

LC–MS raw data were pre-processed for peak extraction, correction, metabolite identification, and annotation, followed by quality control (total ion chromatogram [TIC] overlap, coefficient of variation [CV] distribution plots, and CV filtering). High-quality data were then assessed by principal component analysis (PCA), clustering analysis, and repeatability correlation. Finally, statistical analysis (univariate and multivariate) was performed for functional prediction and interpretation of sample metabolites.

### 2.5 Statistical analysis

SPSS 27.0 (IBM Corp., USA) was used to perform one-way analysis of variance (ANOVA) and Duncan’s multiple range test. Adonis, namely PERMANOVA (Permutational multivariate analysis of variance), conducts multivariate analysis of variance with multiple factors. Differentially accumulated metabolites (DAMs) were identified based on two criteria: Variable Importance in Projection (VIP) score ≥ 1 and absolute value of log2(fold change) ≥ 1. Graphs were prepared using Origin 2024 (OriginLab Corp., USA).

## 3 Result

### 3.1 Effects of nitrogen source and rootstock on plant growth

**Figure 1:**
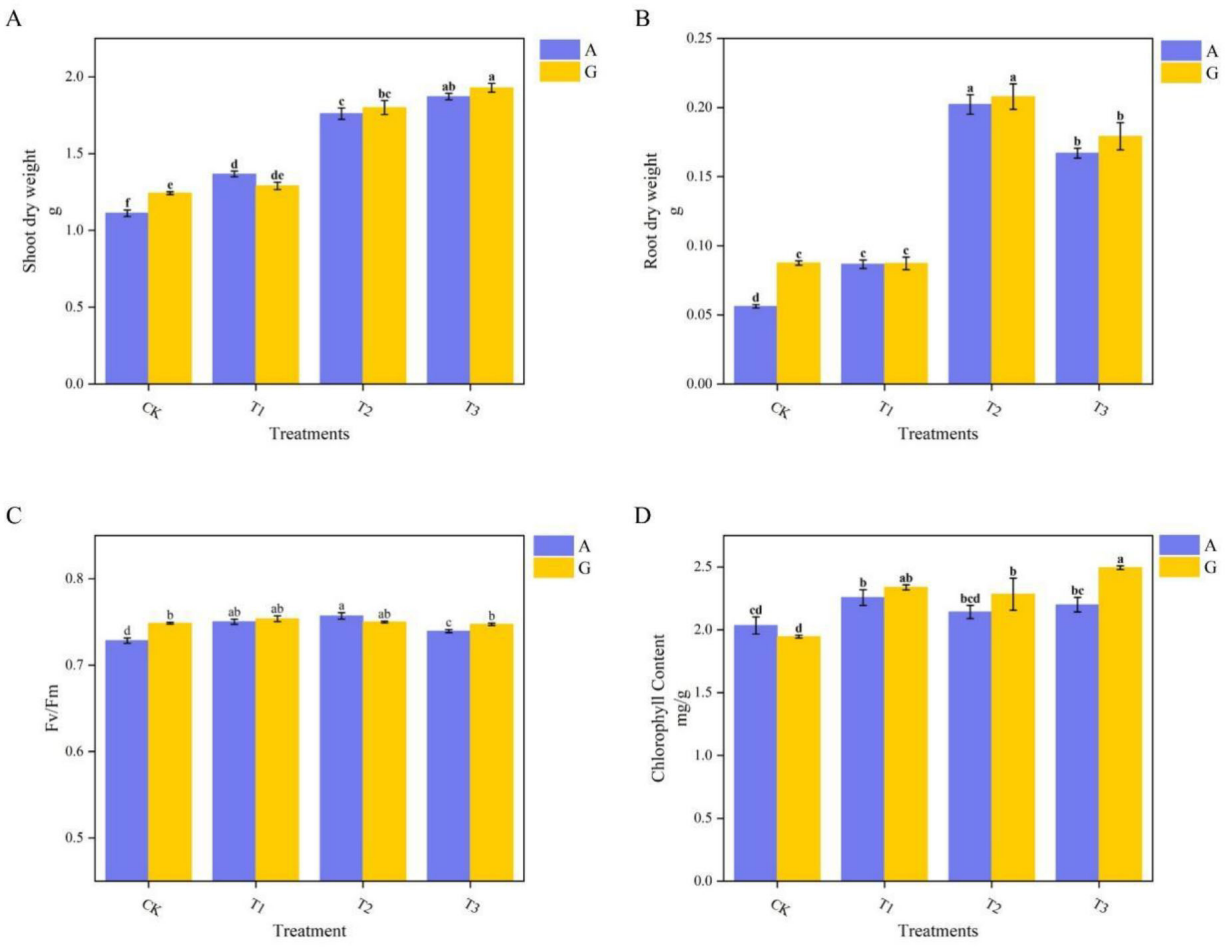
Growth parameters of grafted watermelon under different nitrogen treatments. **(A)** Aboveground dry weight; **(B)** Underground dry weight; **(C)** Chlorophyll fluorescence parameters (PSII maximum photochemical quantum yield, Fv/Fm); **(D)** Chlorophyll content.

The effects of the two nitrogen sources on grafted watermelon growth are illustrated in **Figure 1**. GCK exhibited a significant increase in both aboveground and belowground biomass compared to ACK, whereas no notable differences were observed among the other three rootstocks between the two nitrogen sources. Significant differences in biomass were found among the different grafting treatments using the same nitrogen source, with aboveground and belowground biomass increases ranging from 3.8% to 68.26% and 54.27% to 259.79%, respectively (**Figures 1A, B**). The T3 treatment recorded the highest aboveground biomass at 1.929 g, whereas T2 had the highest belowground biomass at 0.208 g.

Under glycine treatment, no significant differences in the maximum quantum yield of PSII photochemistry (Fv/Fm) were observed among the various grafted rootstocks, with all values approximately 0.75, which is within the normal physiological range. In contrast, under ammonium nitrate treatment, there were no differences between the T1 and T2 grafting treatments, whereas CK and T3 exhibited significant declines in Fv/Fm, with values of 0.728 and 0.739, respectively. Compared with the glycine treatment, T3 and CK displayed significant declines in Fv/Fm by 2.7% and 1.1% under ammonium nitrate (**Figure 1C**).

For chlorophyll content, significant changes were noted between ACK and the AT1 and AT3 treatments under the same nitrogen conditions, whereas no differences were observed among the other grafting treatments. GT3 had the highest chlorophyll content at 2.495 mg/g, whereas GCK showed the lowest at 1.947 mg/g. Across nitrogen types, no significant differences in chlorophyll content were found among the other three grafting treatments; however, GT3 exhibited chlorophyll content that was 11.8% higher than that under ammonium nitrate (**Figure 1D**).

According to **Table 3**, Adonis analysis was conducted on the above four plant growth indicators. The nitrogen source did not show significant differences, while the types of rootstocks showed extremely significant differences.

**Table 3:** Adonis analysis of plant growth under different nitrogen sources and rootstocks; SumsOfSqs, Sum of Squares; MeanSqs, Mean square; F.Model, F value.

Treatments	SumsOfSqs	MeanSqs	F.Model	R^2^	P
Nitrogen source Stock type	0 (0.001) 0.037 (0.002)	0 (0.0003) 0.0123 (0.0003)	2.271 54.994	0.362 (0.638) 0.954 (0.046)	0.2 0.001

### 3.2 Effects of nitrogen source and rootstock on soil enzyme activities

**Figure 2:**
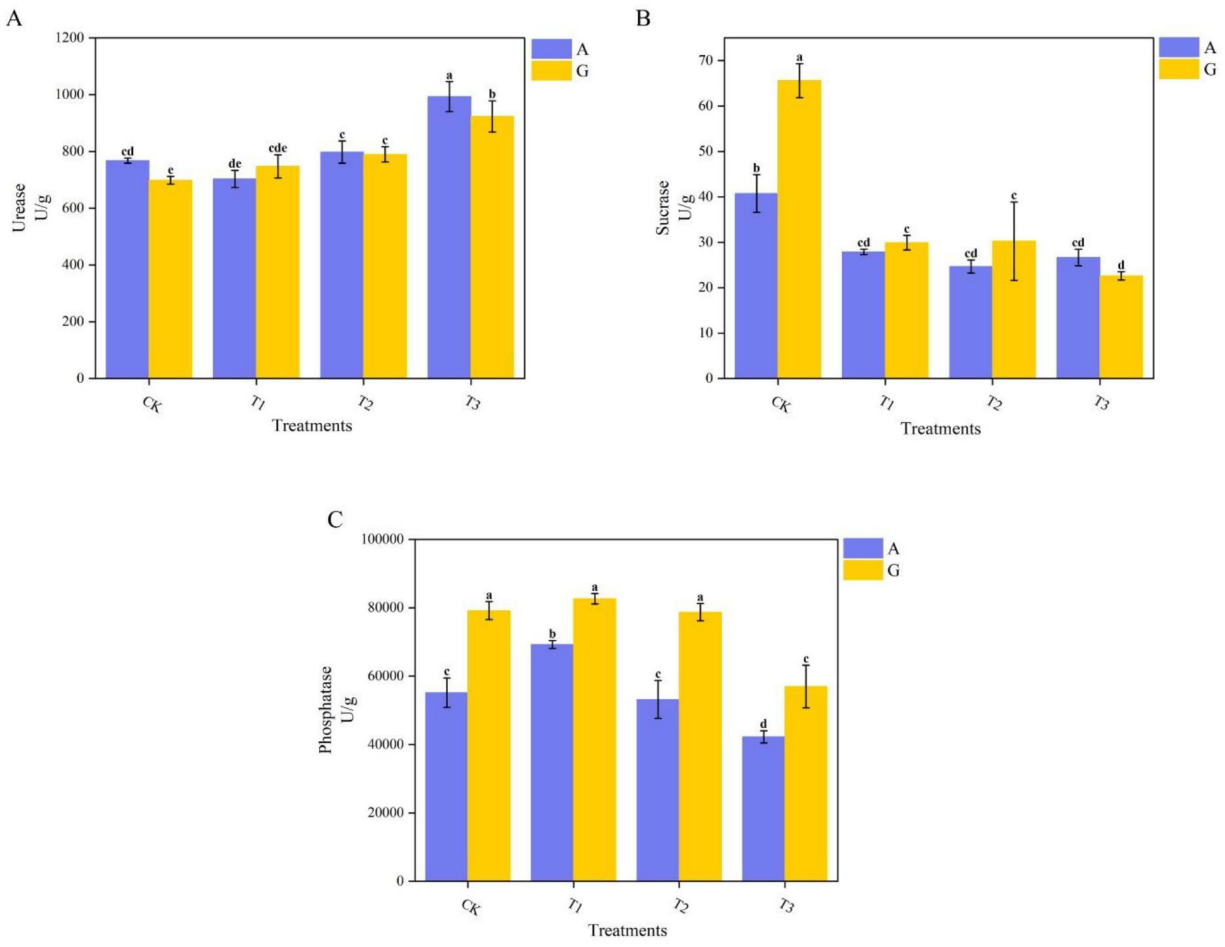
Enzyme activity in the rhizosphere. **(A)** Urease (S-UE); **(B)** Sucrase (S-SC); **(C)** Acid phosphatase (S-AP). Error bars represent standard errors of the mean. Color bars indicate nitrogen source: Ammonium Nitrate (blue), Glycine (yellow).

Soil urease (S-UE) activity (**Figure 2A**) was highest in AT3 soil, reaching 993.23 U/g, and both T3 treatments (across nitrogen sources) demonstrated higher enzyme activities than the other grafting treatments. The lowest urease activity was recorded in GCK soil (698.39 U/g). Urease activities in GCK and GT3 soils were significantly reduced by 9.04% and 7.03%, respectively, compared with ACK and AT3. No significant differences were found between T1 and T2 for either nitrogen source.

For S-SC and acid phosphatase (S-AP) activities (**Figures 2B, C**), enzyme activities were generally higher in glycine treatments compared to ammonium nitrate. Specifically, CK exhibited higher S-SC activity than the other grafting treatments, with the highest activity observed in GCK at 65.60 U/g, representing a significant increase of 61.01% over ammonium nitrate treatment. GT3 had the lowest S-SC activity. S-AP activity (**Figure 2C**) differed significantly (P < 0.05) among nitrogen levels, with GT1 showing the highest activity and AT3 the lowest, at 82,638.71 U/g and 42,239.21 U/g, respectively. Among the top three grafting treatments in the glycine group, no significant differences were found, nor between ACK and AT2. T3 exhibited the lowest S-AP activity across both nitrogen sources.

According to **Table 4**, Adonis analysis was conducted on the enzymatic activities of the above three types of soil enzymes. The nitrogen source did not show significant differences, while the type of rootstock showed extremely significant differences.

**Table 4:** Adonis analysis of soil enzyme activities under different nitrogen sources and rootstocks; SumsOfSqs, Sum of Squares; MeanSqs, Mean square; F.Model: F value.

Treatments	SumsOfSqs	MeanSqs	F.Model	R^2^	P
Nitrogen source Stock type	0.033 (0.001) 0.087 (0.003)	0.033 (0.0003) 0.029 (0.0004)	151.457 76.241	0.974 (0.026) 0.966 (0.034)	0.1 0.001

### 3.3 Nitrogen source and rootstock effects on rhizosphere bacterial community composition

**Figure 3:**
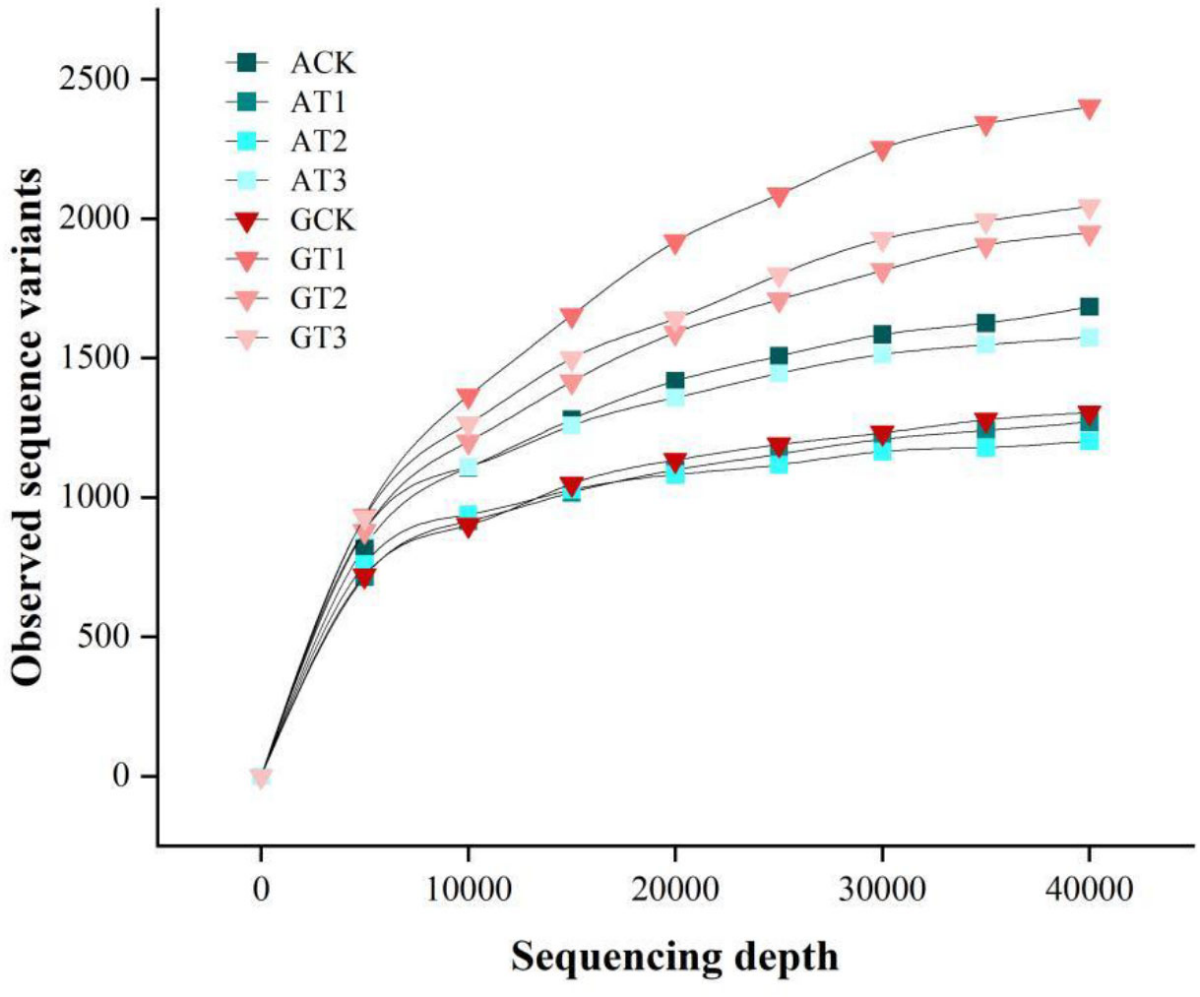
Rarefaction curves of ASV numbers obtained using the Deblur plugin at different non-chimeric read counts (data are randomly subsampled to demonstrate increasing sequence numbers).

Analysis of the rhizosphere bacterial communities from all treatments yielded a total of 2,540,622 high-quality sequences, with an average of 105,859 reads per sample (range: 64,765–119,919 reads), resulting in the identification of 12,790 ASVs. Diversity indices were subsequently calculated. The rarefaction curve (**Figure 3**) showed that at sequencing depths between 5,000 and 40,000 reads, the curve plateaued, indicating sufficient sequencing coverage to capture the majority of sample diversity. These results suggest that the sequencing data robustly represents the ASV diversity in the soil samples, with specific ASV counts detailed in **Table 5**.

**Table 5:** ASV numbers under different grafting conditions and nitrogen sources.

Nitrogen	A				G			
Treatment	CK	T1	T2	T3	CK	T1	T2	T3
ASV numbers	1791	1355	1253	1666	1373	2517	2120	2202

**Figure 4:**
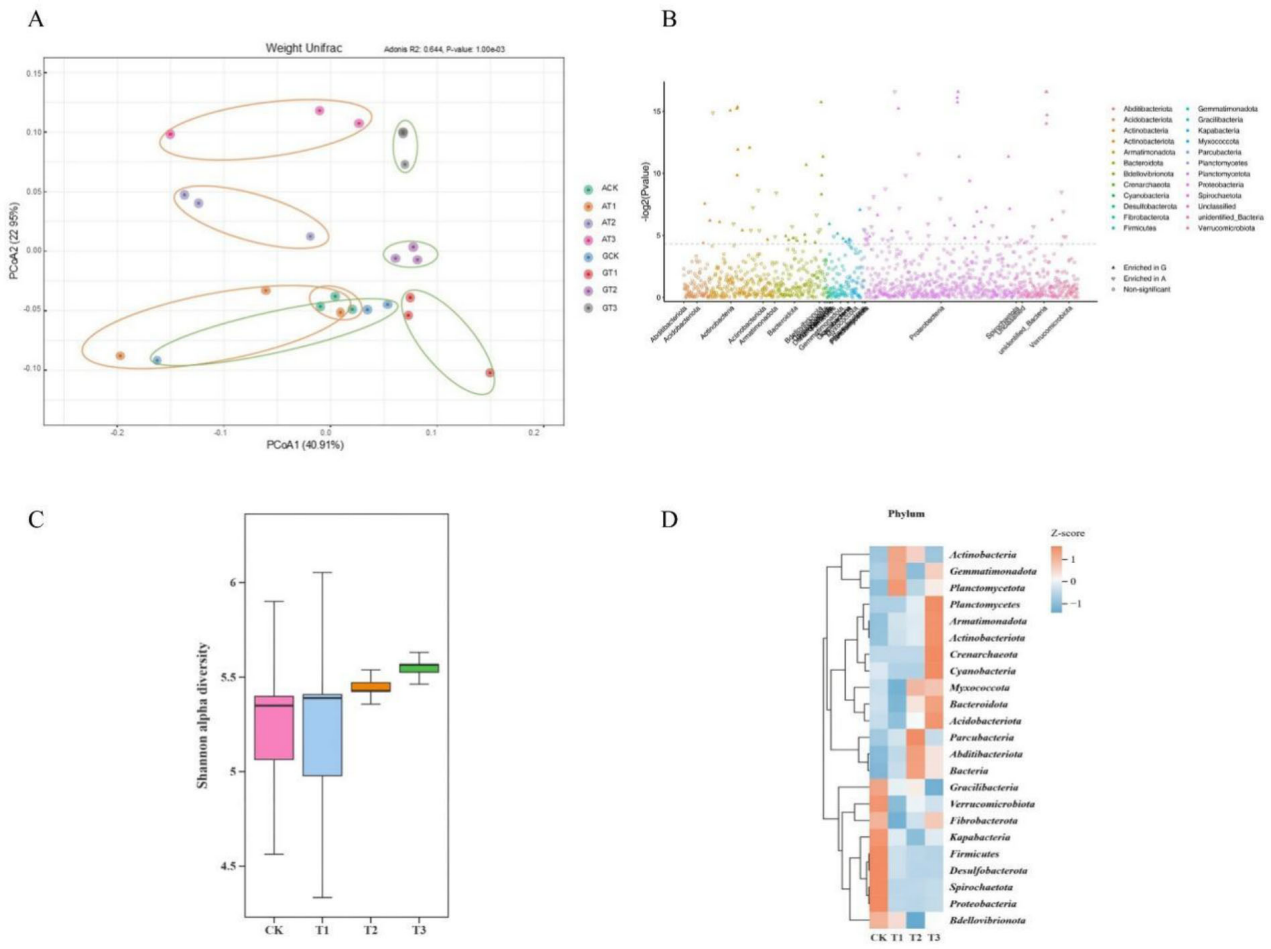
Bacterial community composition in rhizosphere soils under glycine versus ammonium nitrate. **(A)** PCoA of ASVs; **(B)** Manhattan plot highlighting enriched rhizobacterial phyla among differential ASVs (triangles denote differential ASVs, circles denote non-significant ASVs, P < 0.05, Wilcoxon rank-sum test); **(C)** Shannon index of α−diversity; **(D)** Heatmap of the relative abundance of differential ASVs among rootstocks under glycine application. The heatmap uses normalized z-scores for relative ASV abundance.

Further analysis revealed the diversity and structure of the bacterial community across treatments (**Figure 4**). Principal coordinate analysis (PCoA) of Weighted UniFrac distances (**Figure 4A**; adonis, R² = 0.644, P = 0.001) explained over 50% of the total variance, demonstrating the reliability of the data and the significant effect of glycine substitution for ammonium nitrate. The Manhattan plot (**Figure 4B**) of G treatment samples highlighted enrichment of several phyla, including *Acidobacteriota, Actinobacteria, Armatimonadota, Bdellovibrionota, Gemmatimonadota, Myxococcota, Planctomycetota, Proteobacteria, and Bacteria.*

Within the G treatment, no significant differences in the Shannon index were observed among rootstocks (**Figure 4C**). However, **Table 3** indicates that GCK exhibited a significantly lower ASV count than the other rootstock treatments, with GT1 exhibiting the highest count (2,517). Phylum-level heatmap analysis (**Figure 4D**) showed rootstock-specific enrichment: GCK (*Proteobacteria, Spirochaetota, Desulfobacterota, Firmicutes*, etc.), GT1 (*Planctomycetota*), GT2 (*Parcubacteri*a), and GT3 (*Actinobacteriota, Acidobacteriota, Armatimonadota, Crenarchaeota, Cyanobacteria, Planctomycetota*, etc.).

**Figure 5:**
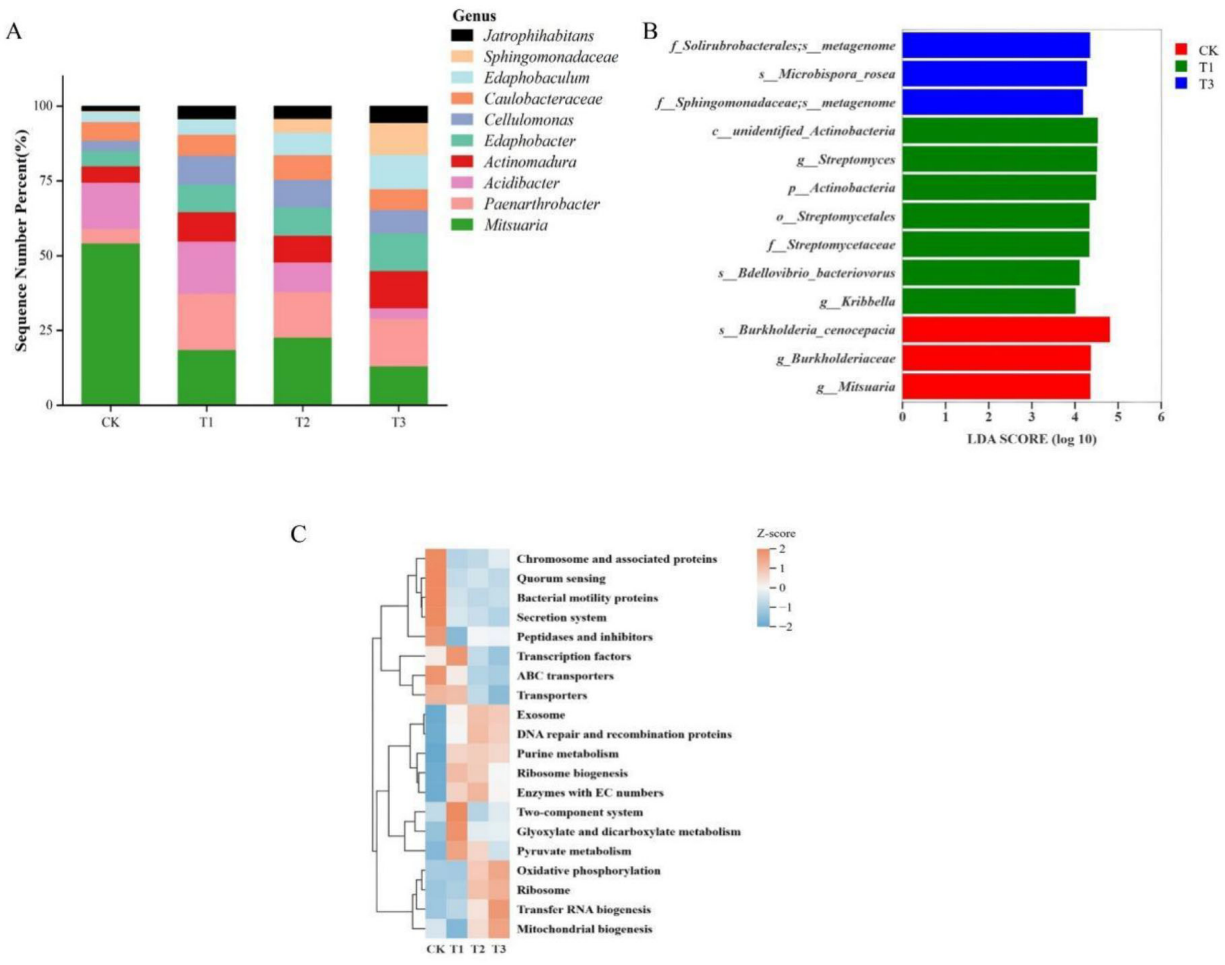
Differences in rhizosphere bacteria among rootstocks after glycine application. **(A)** Top 10 genera by relative abundance in each rootstock; **(B)** Differential PICRUSt functional gene predictions; **(C)** Heatmap of predicted functional pathways.

Genus-level differential analysis was performed for each rootstock, selecting the ten most abundant genera (**Figure 5A**). *Mitsuaria* showed a relative abundance exceeding 50% in CK, whereas *Acidibacter* was more abundant in CK and T1. *Paenarthrobacter* was more prevalent in T1, T2, and T3. *Edaphobaculum* and *Sphingomonadaceae* reached their highest relative abundance in T3, with *Sphingomonadaceae* nearly absent in both watermelon rootstocks. LEfSe analysis identified significant differences in predicted functional gene profiles among rootstock groups, with T1 possessing the most differentially abundant genes and T2 exhibiting none (**Figure 5B**). Clustering analysis of the top 20 KEGG pathways (**Figure 5C**) showed that ABC transporters and secretion systems were enriched in CK, whereas the two-component system, glyoxylate and dicarboxylate metabolism, pyruvate metabolism, and transcription factors were enriched in T1. Oxidative phosphorylation, tRNA biogenesis, and mitochondrial biogenesis were enriched in T3. Purine metabolism was variably expressed in T1, T2, and T3.

**Figure 6:**
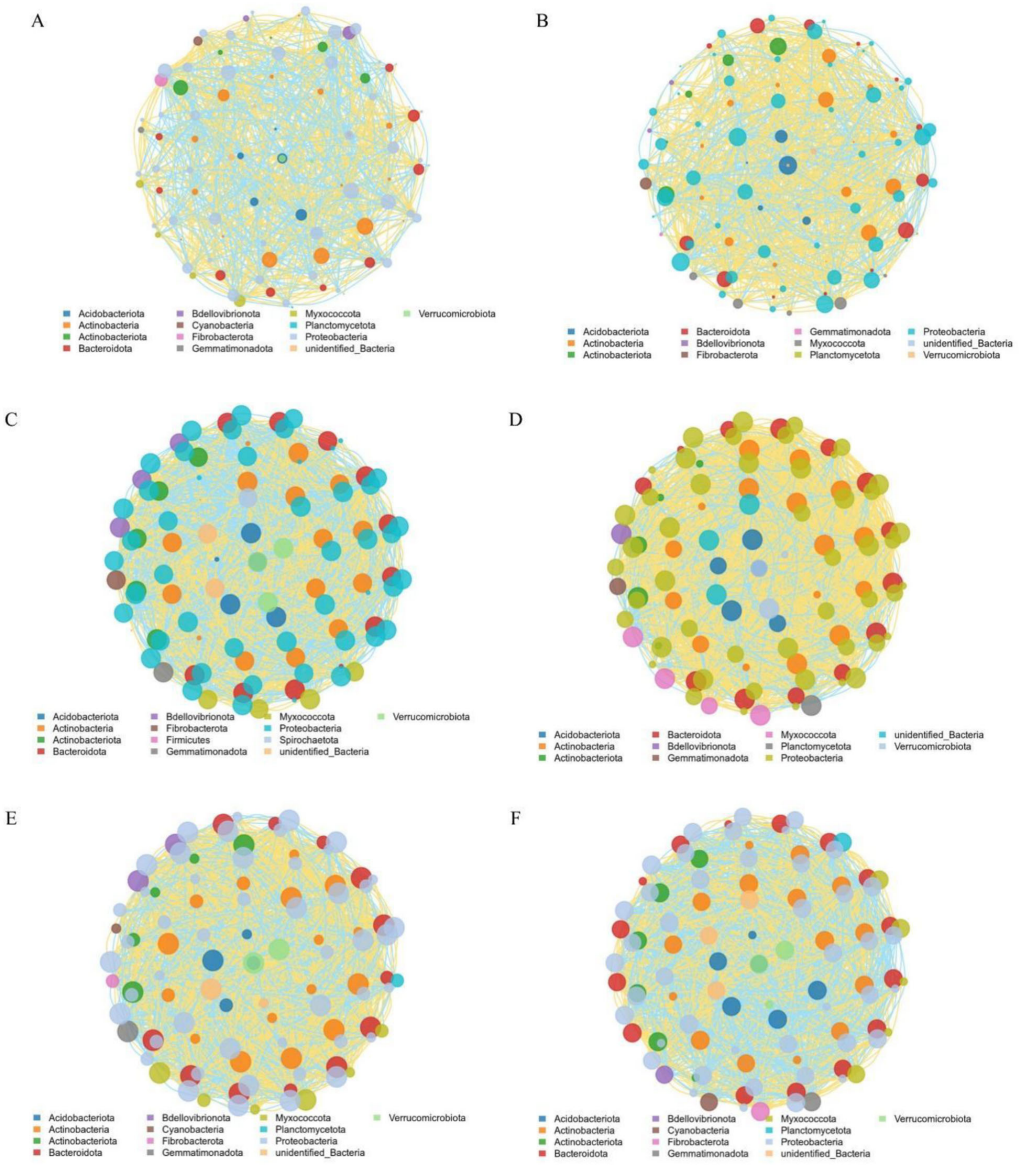
Co-occurrence networks of the top 100 rhizosphere bacterial genera under different nitrogen forms and rootstocks. **(A)** Ammonium nitrate; **(B)** Glycine; **(C–F)** Rootstock-specific networks. Nodes represent genera, colored by phylum; node size corresponds to connection degree; **(C)**: GCK, **(D)**: GT1, **(E)**: GT2, **(F)**: GT3.

Network analysis, based on significant genus-level correlations (Spearman’s correlation, P < 0.05) (**Figure 6**), was performed to assess topological network properties and potential key associations in the rhizosphere community in response to nitrogen form (**Figures 6A, B**). Both node connectivity and network density were lower under ammonium nitrate than under glycine, indicating a less complex network structure in A compared to G (**Table 6**).

**Table 6:** Topological characteristics of bacterial co-occurrence networks under different nitrogen sources.

Treatments	Average degree	Edges	Network density	Positive correlations
A G	15.45 17.68	757 884	0.1593 0.1786	52.05% 61.54%

Subsequently, we performed an Adonis analysis to test the effects of nitrogen source and rootstock as the two factors (**Table 7**). Nitrogen sources showed no significant difference, but rootstock type had a highly significant effect.

**Table 7:** Adonis analysis of different nitrogen sources and rootstocks; SumsOfSqs, Sum of Squares; MeanSqs, Mean square; F.Model, F value.

Treatments	SumsOfSqs	MeanSqs	F.Model	R^2^	P
Nitrogen source Stock type	0.011 (0.006) 0.757 (0.017)	0.011 (0.002) 0.252 (0.002)	7.726 118.985	0.659 (0.341) 0.978 (0.022)	0.1 0.001

Within the glycine treatment (**Figures 6C–F**), CK displayed the highest average degree, node interconnectivity, and network density. The positive correlation ratio in CK was 50.18%, whereas T1 showed the highest ratio at 69.81%, representing a 27–40% increase over the other rootstocks. T2 and T3 exhibited highly similar patterns, with T2 displaying a slightly higher positive correlation ratio (**Table 8**).

**Table 8:** Topological characteristics of bacterial co-occurrence networks under different rootstocks.

Treatments	Average degree	Edges	Network density	Positive correlations
GCK GT1 GT2 GT3	38.78 37.16 35.88 35.88	1939 1858 1794 1794	0.3917 0.3754 0.3624 0.3624	50.18% 69.81% 54.96% 49.94%

### 3.4 Metabolite profiles of roots from different grafted rootstocks

**Figure 7:**
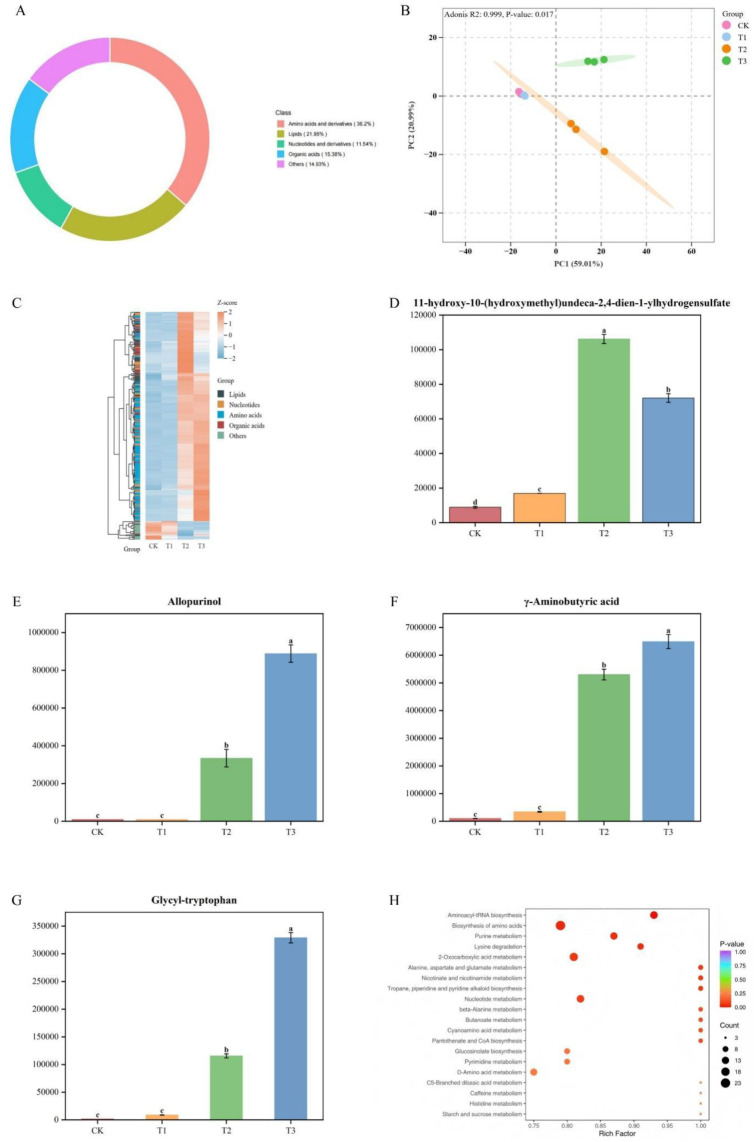
**(A)** Classification of root exudate compounds; **(B)** PCA of root exudates under glycine; **(C)** Hierarchical clustering of differential metabolites among rootstocks; **(D–G)** Representative metabolite concentrations in the four rootstocks; **(H)** Enriched metabolic pathways of root exudates. Each dot in **(H)** represents a metabolic pathway, with size reflecting the degree of change.

UPLC–MS/MS analysis identified 442 compounds in root exudates across all samples, with amino acids constituting the most abundant class (36.20%), followed by lipids (21.95%), organic acids (15.38%), and nucleotides (11.54%) (**Figure 7A**).

Based on differential metabolite analysis among rootstocks under glycine treatment, 261 significant metabolites were identified (VIP > 1 and P < 0.05). PCA (**Figure 7B**) revealed that the first two principal components accounted for 80% of the variance in root exudate composition among the four rootstocks. The results indicated minimal differences between CK and T1, whereas T2 and T3 displayed pronounced metabolic divergence from the other treatments. Hierarchical clustering (**Figure 7C**) confirmed this pattern, with T3 exhibiting the largest number of differential metabolites and T1 the fewest. The 26 metabolites with the smallest P-values (top 10% of 261; **Table 9**) were defined as highly significant. Of these, four representative metabolites—Glycyl-tryptophan, γ-aminobutyric acid, Allopurinol, and 11-hydroxy-10-(hydroxymethyl) undeca-2,4-dien-1-yl hydrogen sulfate—were selected for comparative analysis (**Figures 7D–G**). Glycyl-tryptophan, γ-aminobutyric acid, and Allopurinol were significantly more abundant in T3 than in other rootstocks, with no significant difference between the two watermelon rootstocks. In contrast, 11-hydroxy-10-(hydroxymethyl) undeca-2,4-dien-1-yl hydrogen sulfate was most abundant in T2, and CK had the lowest levels for all four metabolites.

**Table 9:** Top 26 (top 10% of 261) metabolites with the most significant differences.

Compound	Classification	GCK	GT1	GT2	GT3
Glycyl-tryptophan 11-hydroxy-10-(hydroxymethyl)undeca-2,4-dien-1-yl hydrogen sulfate L-Glutamine γ-Aminobutyric acid 4-hydroxy-L-Isoleucine Asn-Ile N,N-Dimethylglycine Methyl 3-aminopropanoate Leu-Asp L-Proline* Meso-Erythritol N-Glycyl-L-leucine Prolylproline L-Lysine L-threo-3-Methylaspartate L-Glutamic acid Hydroxy ricinoleic acid Allopurinol N-Ethylglycine Guanine NG,NG-Dimethyl-L-arginine Val-Pro N5-(1-Iminoethyl)-L-ornithine Isoguanine Cytidine 5’-monophosphate(Cytidylic acid) 2-Aminoisobutyric acid	Amino acids Lipids Amino acids Organic acids Amino acids Amino acids Amino acids Amino acids Amino acids Amino acids Others Amino acids Amino acids Amino acids Amino acids Amino acids Lipids Nucleotides Amino acids Nucleotides Amino acids Amino acids Amino acids Nucleotides Nucleotides Organic acids	1650.368343 8902.161833 144603.6316 102969.3518 196116.4371 2832.3488 58523.98011 24368.67281 2879.6352 202707.3583 2992335.1 8667.2662 12791.29269 86287.25233 5837.174278 490945.0218 530.9099429 9357.149 58444.0829 4304.1775 33472.26583 5829.2 15162.64525 1089.90155 58213.6284 65436.863	8864.335151 16939.07513 556316.5248 343882.0059 185571.8587 2832.3488 141987.2504 92853.46298 18419.02014 730110.3728 2951617.255 51646.72318 31591.22187 827426.5545 7429.279178 468767.7722 4412.415939 9357.149 168287.1027 37680.02383 56178.21896 13601.46667 24487.30905 9717.734333 33395.87853 159754.4589	115578.6191 106174.53 4095526.188 5302294.047 2119721.72 79373.6858 710711.1755 528742.5771 136429.2599 6330837.34 17071.30216 1723725.052 230286.0513 4036758.59 92216.56577 4909625.811 27313.41563 334213.4554 671135.9583 582037.643 232133.0358 309954.3019 93085.95169 191734.8533 290862.1967 707849.3003	329020.5223 72060.61789 12336444.99 6493399.874 2902948.652 446727.7015 1554309.056 1031385.473 866788.8952 11755903.99 1465749.734 4917645.138 455197.4181 11148917.55 121432.3085 6892495.233 11345.36983 888484.5759 1497463.874 2729484.703 479258.1182 999047.8751 107880.4579 1066024.079 450009.447 1582817.324

* Derivatives of a certain type, generally referring to structural analogues or derivatives based on L-Proline

Pathway enrichment analysis of root exudates under glycine treatment identified several significantly enriched metabolic pathways, including aminoacyl-tRNA biosynthesis, biosynthesis of amino acids, purine metabolism, lysine degradation, and 2-oxocarboxylic acid metabolism (**Figure 7H**; Wilcoxon test, p < 0.05).

### 3.5 Correlation between rhizosphere bacteria and root exudates

**Figure 8:**
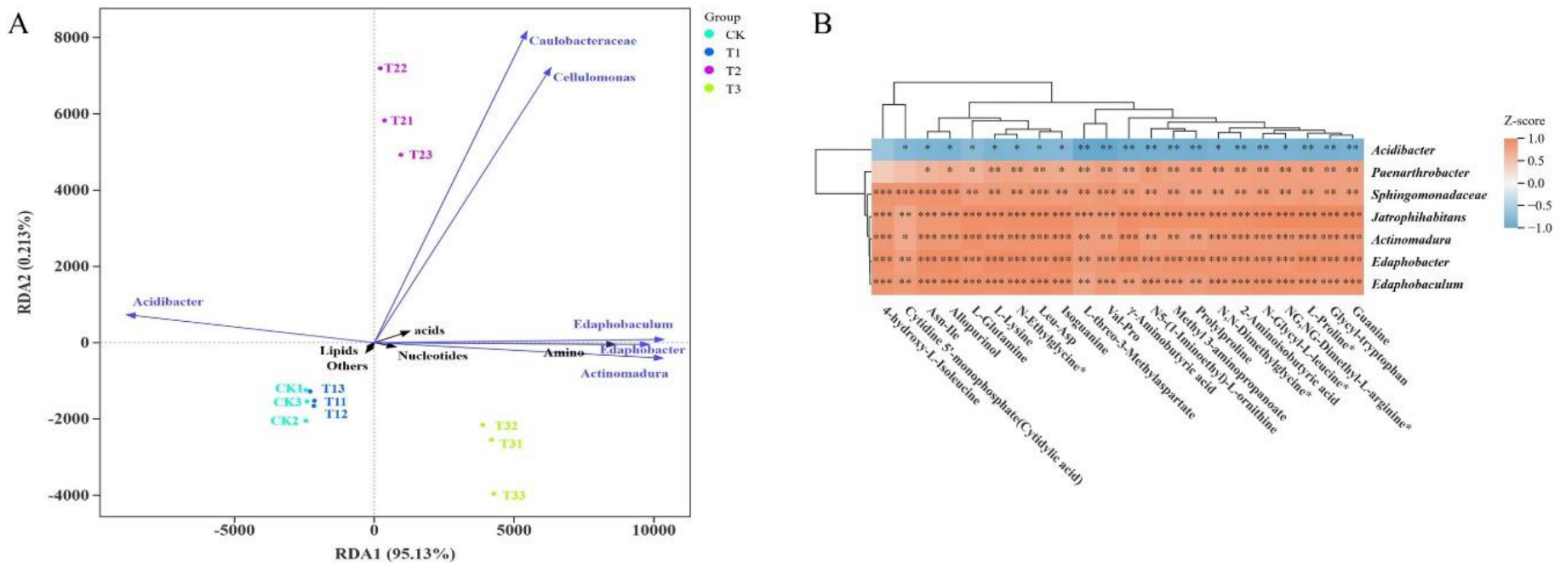
Correlation analysis of differential bacteria and metabolites among different rootstocks under glycine. **(A)** RDA; an angle less than 90° between two variables indicates a positive correlation, whereas an angle greater than 90° indicates a negative correlation. **(B)** Correlation heatmap of microorganisms and metabolites across four rootstocks.

Redundancy analysis (RDA) revealed that the first two RDA axes together explained 95.34% of the variation in the association between metabolites and bacterial taxa, with RDA1 accounting for 95.13% (**Figure 8A**), indicating that RDA1 is the primary dimension distinguishing the association between metabolites and bacterial communities across rootstocks. Among all metabolites, lipids, nucleotides, and amino acids had substantial effects on the composition of rhizosphere bacterial genera, suggesting that these metabolite classes are key regulators of microbial communities through rootstock-mediated secretion. Acidibacter was positively correlated with lipids but negatively correlated with most other metabolites. Conversely, *Edaphobaculum*, *Edaphobacter*, *Actinomadura*, and several metabolite classes—including amino acids, nucleotides, and organic acids—were strongly positively correlated. Caulobacteraceae and Cellulomonas also showed positive associations. These results suggest that *Edaphobacter* and *Actinomadura* are core genera associated with amino acid and nucleotide regulation, driving the metabolic characteristics of bacteria across different rootstocks. Correlation heatmap analysis (**Figure 8B**) identified significant associations between 7 bacterial taxa and 22 metabolites. *Acidibacter* showed negative correlations with 21 metabolites (11 highly significant). *Edaphobacter* and *Actinomadura* were extremely significantly positively correlated with 20 and 16 metabolites (excluding lipids), respectively. *Sphingomonadaceae*, *Jatrophihabitans*, *Edaphobaculum*, and *Paenarthrobacter* were positively correlated with all 22 metabolites, with highly significant positive correlations for 6, 21, 18, and 20 metabolites, respectively.

Collectively, these analyses indicate that primary metabolic pathways promote the abundance of *Edaphobacter* and *Actinomadura*, and that four other genera also play critical roles in the positive regulation of metabolite dynamics. Under glycine administration, rootstocks selectively regulate metabolite distribution, which in turn shapes the structure of the rhizosphere microbial community.

## 4 Discussion

### 4.1 Glycine modulates soil enzymes and rootstock performance

Soil enzyme activity is frequently utilized as an indicator of microbial growth. S-UE plays a critical role in the nitrogen cycle (Tamaddon et al., 2020), and provides nitrogen nutrients to plants (Lee et al., 2021). The higher S-UE in treatment A compared to treatment G may be attributed to the rapid conversion of ammonium nitrogen and the subsequent release of nitrogen, which stimulates the production of urease or urease-associated microorganisms. Previous research has shown that many tested species preferentially absorb inorganic nitrogen (Zhuang et al., 2020). The application of two forms of nitrogen resulted in different microbial community structures, leading to varied effects on soil enzymatic activities. Additionally, the extensive root system and enhanced nitrogen metabolism capacity of pumpkin resulted in the highest enzyme activity observed. S-SC can convert oligosaccharides in soil into monosaccharides needed by plants and facilitate the circulation of organic nitrogen in soil (Farooq et al., 2021). Although S-SC activity in treatment G was greater than that in treatment A, the difference was not significant. The application of glycine provided not only a nitrogen source but also a carbon source, thereby altering the C/N ratio and affecting the production of sucrase. This is consistent with the findings of (Zeng et al., 2024), which demonstrated that glycine promotes sucrase activity. The higher enzyme activity in CK compared to the other grafting treatments may be attributed to compatibility issues between the scion and the rootstock, as (Wang et al., 2024) reported that heterografting can suppress the gene expression of enzymes related to sucrose metabolism. Like S-SC, the application of glycine supplied both carbon and nitrogen. Furthermore, glycine reduces the pH of the substrate, leading to lower S-AP activity in ammonium nitrate treatments. Therefore, the application of glycine and the combination of different rootstocks significantly changed the biological activity of soil enzymes, and the interaction among these enzymes was also a key factor in modifying the soil environment (Zhou et al., 2025).

Regarding biomass, bottle gourd and pumpkin demonstrated advantages, likely due to their robust root systems (Sallaku et al., 2022) or their strong adaptability, which supports better growth of watermelon (Xiong et al., 2021), ultimately increasing both aboveground and underground biomass. Overall, Fv/Fm and chlorophyll content in certain grafting treatments of treatment A significantly decreased compared to the normal levels in treatment G. The application of exogenous glycine promotes the production of endogenous compounds in plants, such as the glycine-rich protein-encoding gene *GhGRPL*; its upregulation enhances plant stress tolerance by thickening secondary cell walls (Yu et al., 2024) This finding is consistent with (Xu et al., 2022), which demonstrated that glycine enhances plant tolerance, helping maintain various physiological parameters at normal levels.

Based on the observed changes in S-UE, S-SC, and S-AP activities induced by glycine, suppose that glycine might can reduce the demand for nitrogen metabolism, alter the nutrient limitation pattern (Liu et al., 2022). The coordinated optimization of the photosynthetic system and the increase in plant biomass following glycine application indicate that glycine can enable efficient conversion of photosynthetic carbon assimilation to biomass by increasing light capture capacity and maintaining photochemical reaction efficiency. Although glycine has advantages as described above, the differences among rootstock types are much greater than those among nitrogen sources. Therefore, the combination of rootstock and glycine is particularly important (**Tables 3**, **4**).

### 4.2 Glycine modulates rootstock-specific microbial networks

Plant-associated microbial communities play a critical role in plant health and environmental adaptation in natural ecosystems (Trivedi et al., 2021). The exogenous application of different nitrogen sources (Gallart et al., 2018) can shape diverse microbial community structures to help plants cope with environmental stresses or biotic challenges (de Vries et al., 2020; Gao et al., 2024b). IIn this study, glycine was applied as a substitute for ammonium nitrate, and rootstock replacement via grafting further modified rhizosphere microbes to enhance plant resilience. These findings support the optimization of watermelon cultivation practices and contribute to agricultural environmental sustainability. At all rarefied sampling depths (**Table 5**), except for the GCK treatment, glycine treatments exhibited higher ASVs, indicating that the application of glycine has the potential to promote bacterial diversity. Significant changes in β-diversity were observed in the rhizosphere (**Figure 4A**). Differential analysis using a Manhattan plot (**Figure 4B**) of bacterial communities shaped by the two nitrogen sources revealed that glycine substitution led to the enrichment of several phyla. For example, in GCK, *Proteobacteria* (Yang et al., 2024) exhibit rapid carbon turnover; in GT1, *Planctomycetota* (Klimek et al., 2024) and in GT2, *Parcubacteria* (Fujii et al., 2022) demonstrate graded utilization of labile and recalcitrant carbon. In GT3, *Actinobacteriota* and *Acidobacteriota* (Camargo-Montoya et al., 2025; Chen et al., 2021; Palaniyandi et al., 2013) enhance environmental adaptation and participate in biological nitrogen fixation. Previous studies (Luo et al., 2018) have demonstrated that different forms of nitrogen influence unique soil microbial compositions. Similarly, (Morais et al., 2024) found that grafting can alter a plant’s rhizosphere microbiome. Therefore, four common grafting combinations—Watermelon/Watermelon, Watermelon/Wild watermelon, Watermelon/Bottle gourd, and Watermelon/Pumpkin—were used to determine optimal scion-rootstock utilization for watermelon.

For the G treatment analysis, no significant difference was observed in the Shannon index (**Figure 4C**), although numerical differences were present. This may be due to the long-term response required for microbial communities to adapt to environmental changes (Davis et al., 2005), suggesting that the duration of this study may have been insufficient to capture such shifts. Cluster heatmap analysis of the four rootstocks (**Figure 4D**), together with differential analysis between the two nitrogen sources (**Figure 4B**), revealed that the significant phyla influenced by glycine were primarily present in CK, T1, and T3, as shown by the LefSe analysis (**Figure 5B**) of significantly different functional genes in the four rootstock microbiomes; T2 treatment exhibited no differential genes. The top ten differential microbes from the four rootstocks were analyzed. *Mitsuaria* was enriched by over 50% in CK, whereas the relative abundance of *Paenarthrobacter* was significantly lower than in other treatments. *Sphingomonadaceae* was enriched in the non-watermelon rootstocks T2 and T3. CK plants may recruit more *Mitsuaria* due to their weaker root systems, which supports nutrient absorption and helps plants withstand environmental challenges (Huang et al., 2017; Marian et al., 2018). Meanwhile, *Paenarthrobacter* has been identified as a degrader of organic matter, enhancing plant growth, productivity, and antioxidative capacity (Ghasemi et al., 2018; Liu et al., 2024). The recruitment of *Mitsuaria* in CK may have taken precedence; as shown in **Figure 5B**, the *Mitsuaria* gene in CK is significantly expressed, partially replacing the role of *Paenarthrobacter* and resulting in less enrichment. *Sphingomonadaceae* possess strong degradation capabilities, increasing plant resistance to pathogens and stress (Sultana et al., 2023; Zhou et al., 2023). Functional predictions using PICRUSt revealed significant differences in Level 3 pathways contributed by rootstock bacteria (**Figure 5C**).

The microbial network analysis indicated that substituting ammonium nitrate with glycine increased bacterial interactions (**Figures 6A, B**; **Table 6**). Adonis analysis (**Table 7**) revealed no significant difference between nitrogen sources but a highly significant difference among rootstocks (p < 0.01). Based on these results, we shifted our focus to glycine’s effects on different rootstocks. Comparisons among rootstocks showed that interactions among bacteria in wild watermelon rootstocks were stronger (**Table 8**) and nearly self-compatible, consistent with the findings of (Gu et al., 2024). Correlation analysis of the top 100 bacteria demonstrated that T3 treatment exhibited the highest rate of negative correlations. Studies suggest that ecological competition through negative regulation among bacteria can enhance microbial community stability (de Vries et al., 2020), benefiting plants by competing against external stresses (Hernandez et al., 2021). The high positive correlation ratio of T1 may be due to the abundance of functional genes (**Figure 5B**) and a well-developed two-component system (**Figure 5C**), resulting in enhanced environmental perception and mobilization of the microbial community, revealing a strong synergistic symbiosis induced by the rootstock. The network structure of T3 is similar, but its positive correlation ratio is not high because its energy metabolism (**Figures 5B, C**) is strengthened, leading to intensified resource competition and better buffering capacity under stress. CK has high ABC transporter gene expression (**Figure 5C**) and dominates nutrient competition.

Compared with ammonium nitrate application, although glycine treatments did not increase microbial α-diversity, it did show the specific enrichment of functional groups across different rootstocks. A more detailed analysis and functional prediction of the bacterial genera in each glycine treatment, combined with the bacterial network structure, reflect the influence of rootstocks on the energy distribution and nutrient acquisition strategies of microorganisms. These results, together with the decrease in soil enzyme S-UE activity and the increase in S-SC and S-AP activities, enhanced photosynthesis, and increased biomass. This demonstrates the adaptive division of labor of microorganisms in the soil–plant positive feedback system promoted by glycine application and rootstock optimization.

### 4.3 Rootstock-specific metabolites steer rhizosphere microbiome functions

Root exudates directly regulate the activity, abundance, and functional composition of rhizosphere microbial communities (Wen et al., 2020). Differential metabolite analysis under glycine and ammonium nitrate application identified significant changes in 261 of 442 metabolites. PCA of glycine-treated samples (**Figure 7B**) revealed minimal differences between CK and T1, both watermelon rootstocks, whereas T2 and T3 exhibited pronounced metabolic divergence, suggesting a rootstock effect on root exudate profiles. Hierarchical clustering (**Figure 7C**) further highlighted substantial metabolic differences between watermelon rootstocks and those of gourd and pumpkin. Notably, three of the four most differentiated metabolites were highest in pumpkin rootstock (T3; **Figures 7D–G**), and these metabolites serve defensive, adaptive, or buffering roles. For instance, γ-aminobutyric acid (GABA) not only mitigates stress under adverse conditions but also functions as a signaling molecule (Kaspal et al., 2021). Such organic compounds in root exudates regulate nutrient and energy flows that drive rhizosphere microbial colonization, thereby influencing the structure and dynamics of the microbial community (Iannucci et al., 2017). The results demonstrate that CK and T1 rootstocks show limited metabolic differences (**Figure 7C**), whereas T2 and T3 rootstocks display greater metabolic divergence, with T2’s intensity being lower. The pumpkin rootstock (T3) appears to recruit bacterial taxa such as *Sphingomonadaceae* through the secretion of defense-related metabolites, alleviating stress and activating GABA receptor coordination with the microbiota. This aligns with observed trends in soil enzyme activity, microbial community enrichment, network analysis, and the increase in biomass and maintenance of Fv/Fm.

RDA analysis (**Figure 8A**) confirmed that metabolites from CK, T1, and T3 significantly affected the bacterial community, with T3 showing the strongest metabolite–bacteria interactions. The dominant bacterial genera in these interactions were *Actinomadura* and *Edaphobacter*, which are associated with improved nutrient utilization, metabolic regulation, and stress alleviation (Hamedi and Mohammadipanah, 2015; Hemmat-Jou et al., 2018). Correlation clustering (**Figure 8B**) indicated that oligotrophic *Acidibacter* (Gao et al., 2024a) did not demonstrate a competitive advantage under these conditions, whereas other genera contributed to degradation, growth promotion, nutrient cycling, and stress adaptation (Chen et al., 2023; Gan et al., 2014; Hemmat-Jou et al., 2018; Kruczynska et al., 2023; Liu et al., 2024). Functional pathway predictions (PICRUSt; **Figure 4C**) and metabolite pathway analysis (**Figure 7E**) revealed convergence in purine metabolism, a pathway also enriched in T1 and T3. Glycine likely promotes purine metabolism by acting as a precursor for inosine monophosphate (IMP), a prerequisite for all purine nucleotides.

Both the core RDA1 axis and the correlation heatmap (**Figure 8B**) highlight the interactions among rootstock, microbiota, and metabolites: amino acids, nucleotides, and organic acids are enriched by *Edaphobacter* and *Actinomadura*, whereas lipids specifically promote *Acidibacter*. These findings indicate that rootstock genotype influences the distribution of exudate metabolites, precisely regulating the functional structure of the rhizosphere microbiome. For example, pumpkin rootstock (T3) secretes more amino acids and nucleotides, enriching *Edaphobacter* and *Actinomadura* to reinforce nitrogen cycling and stress resistance. The wild watermelon rootstock, with a highly positively correlated microbial network, enriches *Edaphobacter* and *Actinomadura* and, through lipid-enriched *Acidibacter*, enhances acid adaptability.

Overall, glycine to some extent improved soil enzyme activity, plant biomass, and microbial diversity, supporting the hypothesis that glycine is an effective, eco-friendly nitrogen source combined with different rootstocks for use that promotes sustainability through enrichment of beneficial bacteria and optimization of nutrient cycling. The rootstock-specific structuring of the rhizosphere microbiome via exudate-mediated recruitment and the observed correlations between specific exudate compounds and microbial abundance substantiate the central role of plant–microbe interactions in sustaining the benefits of glycine amendment.

In summary, substituting ammonium nitrate with glycine and selecting suitable scion/rootstock combinations provide new evidence for sustainable agriculture and elucidate the role of root exudates in rhizosphere microbiome assembly. Although associations between metabolites and microorganisms have been established, the complexity of microbial interactions remains to be fully elucidated, and the proposed shift in nutrient limitation patterns requires future validation through stoichiometric analysis of soil chemistry.

## 5 Conclusions

This study deciphered how rootstock-specific microbiome and metabolome remodeling governs glycine substitution efficacy for ammonium nitrate in grafted watermelon systems. The findings demonstrate that glycine application can moderately increase bacterial diversity. It can also enhance soil enzyme activity. Glycine improves nutrient absorption in plants and helps maintain typical plant growth. However, glycine’s effects in these areas are not significantly different from those of ammonium nitrate. Its primary advantage over ammonium nitrate is an environmental benefit. Combining specific rootstocks and scions can enhance glycine’s effectiveness. This combination also allows customization based on cultivation environment and production goals. This work advances crop domestication theory by establishing root exudate chemistry as a tunable factor for rhizosphere engineering and sustainable agricultural management.

## Data Availability

The datasets presented in this study can be found in online repositories. The names of the repository/repositories and accession number(s) can be found below: https://www.ncbi.nlm.nih.gov/, PRJNA1237532.
